# The effect of positive inter-group contact on cooperation: the moderating role of individualism

**DOI:** 10.3389/fpsyg.2024.1323710

**Published:** 2024-03-05

**Authors:** Rikui Xiao, Shuxin Li

**Affiliations:** Department of Sociology, Graduate Institute for Taiwan Studies of Xiamen University, Xiamen University, Xiamen, China

**Keywords:** inter-group contact, cooperation, culture orientation, social distance, individualism

## Abstract

**Introduction:**

The effect of intergroup contact on cooperation is well documented, but little is known about the cultural moderators of this relationship. Contributing to the literature, we examined whether cultural orientation moderates the effect of positive intergroup contact on cooperation and places emphasis on individualism.

**Methods:**

By creating a gamecooperation situation by the trust game paradigm, 322 Taiwanese youth were involved in the study and completed the positive intergroup contact scale, individual-collectivism scale, and social distance scale.

**Results:**

(1) positive intergroup contact effectively promotes cooperative behavior; (2) Taiwanese youth who have closer social distance with mainland youth demonstrate higher levels of cooperative behavior after group interactions than larger social distance; and (3) individualism has a significant moderating role in the relationship between positive inter-group contact and cooperation. The effect of positive inter-group contact on cooperation became stronger in the less individualistic group. The effect of social distance on cooperation became stronger in the less individualistic group.Cultural orientation represented by individualism is proved to be one moderato of the intergroup contact-cooperation relationship.

## 1 Introduction

How to achieve sustainability and effectiveness of intergroup cooperation has been an important point for researchers. Some studies initially explored the occurrence and causes of intergroup cooperation from economic perspectives, such as direct benefits and reciprocity (Trivers, 1971; Lehmann and Keller, 2006; Nowak, 2006). They pointed out that shared interests, benefit exchanges, establishing positive intergroup relations, and enhancing group competitiveness are significant to intergroup cooperative behavior. By promoting the aforementioned factors, intergroup cooperation can be improved. However, research based on economics centered on interests has limitations. Consequently, other researchers started to focus on social psychological factors, including different intergroup contact types, such as direct and imagined contact, and paid attention to social distance. Studies have revealed that contact experiences with out-groups can mitigate intergroup anxiety and negative attitudes, reduce social distance (Brambilla et al., 2012), and promote cooperative intentions and behaviors toward them (Meleady and Seger, 2016). Existing research has emphasized economic rationality, positive intergroup contact, and social distance, as well as their roles in shaping and promoting intergroup cooperation, yet it lacks a cultural dimension.

Culture, as shared values, norms, thinking models, behaviors, and cultural products among members of society (Song, 2018), gives rise to specific interpersonal interaction cognitions within that cultural environment (Blake et al., 2015). It is also an essential factor influencing cooperative behavior. Moreover, Hofstede (1980) classified the psychological and behavioral tendencies formed by individuals in specific cultural environments as cultural beliefs, one of which is individualism–collectivism. In his view, individualism represents a loose social framework where people should only care for themselves and immediate relatives, while collectivism embodies a close-knit social framework (Grossmann and Na, 2014). It is widely accepted that individualism emphasizes independence, uniqueness, and freedom of choice, while collectivism underscores interdependence, social embeddedness, obligations, and loyalty to in-groups, prioritizing collective interests (No et al., 2008; Scott and Marshall, 2009; Van Segbroeck et al., 2008; Varnum and Kitayama, 2011; Zhu et al., 2022). As such, individuals with preferences for individualism or collectivism exhibit different tendencies in cooperative choices: individualism-oriented individuals tend to prioritize benefits in their cooperative decisions, while collectivism-oriented individuals consider their relationship with the cooperative partner more.

Why do different cultural beliefs lead to differences in intergroup cooperation behavior? At the micro level, the self-construction model explains the mechanism of individual cognitive differences in cooperative behavior (Turner et al., 1987; Germani et al., 2020; Kolarova and Ziaran, 2016). This theory suggests that individuals, when constructing themselves and the collective, unconsciously link their behavior to others, giving rise to cooperative behavior. However, due to differences in cultural beliefs, individuals hold completely different perspectives on the relationship between themselves and others. For example, Westerners emphasize individualism and individuality, which belong to the typical independent self-construction, while Easterners emphasize the relationship between themselves and others, which belongs to the typical interdependent self-construction. Therefore, eastern groups tend to cooperate more than western groups. At the macro level, the cultural adaptation theory also provides an explanation (Tajfel and Turner, 1986). Different social groups have gradually formed unique social environments during long-term evolution. For example, East Asian social environments emphasize the interconnection between individuals and advocate the power of the collective, while Western social environments advocate individual autonomy and the power of the individual. However, it should be pointed out that the social environment of a country or region (cultural atmosphere, historical background, and education system) cultivates a specific individualism–collectivism orientation to meet the needs of social development (Campanha et.al., 2011; Desforges et.al., 1991). For example, in countries and regions with a collectivist culture, individuals with a collectivist tendency outnumber those with an individualistic tendency. However, considering that individual factors are important factors influencing individualism–collectivism orientation, individuals growing up in different families and environments have differences in cultural beliefs due to differences in personal experiences, not to mention the changes in cultural beliefs brought about by social changes. It can be seen that individualism and collectivism orientation are one of the main operational methods to define cultural belief differences between groups, and cultural beliefs are influenced by macro and micro factors. More importantly, cultural beliefs are an important factor affecting intergroup cooperation behavior (Ladbury and Hinsz, 2018; Marcus and Le, 2013).

However, there is a lack of in-depth exploration of the effect of intra-group cultural beliefs on intergroup cooperation in existing research. This is probably because previous studies focused on participants within the same cultural belief framework (Mendes et al., 2001; Dixon et al., 2005). More specifically, Dixon et al. (2005) argued that intergroup interactions are influenced by in-group norms and values rather than cultural differences. Ma et al. (2016) pointed out that even within the same cultural background, there are significant differences in individualistic-collectivist orientation among different individuals. For example, Morling and Lamoreaux (2008) compare collectivism and individualism between Eastern and Western countries, and the results show that individualism and collectivism differ significantly not only between East and West but also within different Eastern countries and regions. This suggests that we should focus on the cultural differences between and within groups, that is, when studying cross-border group contact, we should pay attention to the differences in inter-group cooperative behavior that different cultural beliefs may lead to. Therefore, focusing on the role of individualism–collectivism is of great practical value when studying cross-strait intergroup contact.

According to Germani et al. (2021), individualism–collectivism can be scrutinized at both the cultural level, representing the culture of a nation as a whole, and at the individual level, reflecting people’s beliefs about their relationship with others. Individualism–collectivism at the cultural level is conceptually or operationally different from individualism and collectivism at the individual level. Firstly, the social environment of a country, shaped by the cultural atmosphere, historical reasons, and educational patterns, cultivates different orientations in individualism–collectivism to meet different social development needs. It is possible to categorize a country as an individualistic or collectivistic culture (e.g., Hofstede et al., 2010). Even within collectivistic cultures, there are more people with a collectivistic than an individualistic orientation and vice versa. Individualism and collectivism can also be analyzed at the individual level (Triandis, 2001). In other words, individualism and collectivism may be conceptualized and measured as separate, not necessarily opposing, constructs at the individual level. Individual factors likely affect individualism–collectivism (Shavitt et al., 2006; Germani et al., 2021). Therefore, within the same country or region, at the individual level, we can distinguish individuals as being more or less individualistic/collectivistic (individual level).

In particular, Chinese Taiwan is an area where collectivist culture and individualist culture are prevalent, and the cultural orientation of young people in Taiwan is more diversified. Therefore, the role of individualism–collectivism can be ideally studied with Taiwanese youth. Since Taiwanese society has changed from a traditional agricultural type to a modern pluralistic one, traditional and modern values are competing, with modern values gaining ground at a rapid pace in Taiwan (Yang, 1981). Many studies supported the claim. For example, Chiou (2001) tested the individualism–collectivism of 264 Taiwanese students and 254 American students and found that Taiwanese youth scored highly in vertical individualism and horizontal collectivism, even with no significant difference from the U.S. participants. As increasing individualism and decreasing collectivism have become a cultural and psychological change around the globe in recent years (Hamamura, 2012; Manago, 2014), many eastern countries supporting collectivism also experienced a variety in individualism–collectivism (Kashima et al., 2009, 2011). On one hand, traditional cultural education in Taiwanese society places emphasis on cultivating traditional Chinese spirits and virtues, particularly the sense of collectivist responsibility toward family and society (Kong, 2016). This is clearly reflected in events like “Filial Piety Month” in Taiwan. On the other hand, influenced by Western and Japanese cultures, Taiwan has absorbed certain individualistic elements (Chen, 2013). As Taiwan transitions from a traditional agrarian society to a modern and diversified one, modern values that prioritize individual liberation and self-worth, such as the “small happiness” culture, have gained prominence (Zhang and Liu, 2020). Studying Taiwanese youth helps to deeply understand the inter-group contact between cross-strait youth, the young generation of cross-Taiwan strait who are raised in Chinese mainland and Taiwan and grow up under the common cultural heritage of Chinese civilization, yet live in different political and social contexts. It also provides ideas for inter-group contact in eastern regions.

Besides, although intergroup contact has emerged as a pivotal approach for deepening cross-strait communication, the Chinese government has introduced pro-Taiwan policies and built platforms (e.g., cross-strait youth employment and entrepreneurship base) to promote various types of non-governmental and official intergroup contact, regarding Taiwanese youth as family. Mainland youth are also looking forward and hoping to build friendships with Taiwanese youth. However, a lack of institutional support in the Taiwan region poses a significant challenge to the effect of intergroup contact. Furthermore, Taiwan authorities have hindered the exchange activities of Taiwanese youth with Chinese mainland youth through policies. This makes cross-strait contact lack the essential institutional support mentioned by the contact theory. In this context, there are no perfect conditions for interaction, only people who are more suited for contact.

Considering that the essence of cross-strait youth exchange is a form of intergroup contact, this paper takes intergroup contact theory as the primary theoretical framework for this study. Exploring the effect of inter-group contact on Taiwanese youth with different cultural orientations helps to improve cross-strait contact and reveals the role of cultural beliefs on the effect of inter-group contact. Therefore, this study used Taiwanese youth as participants and explored the influence of positive inter-group contact on cooperative behavior and the role of individualism–collectivism in Taiwanese youth.

### 1.1 The effectiveness of inter-group contact in promoting cooperation

Positive inter-group contact refers to the strategy of improving inter-group attitudes through positive and effective contact and interaction (Pettigrew and Tropp, 2006). Everett et al. (2015) study pointed out that “actual cooperation with the other group to achieve mutual welfare” is a sign of positive inter-group relationships. In recent years, numerous studies have examined the impact of inter-group contact on cooperative behavior and found that positive inter-group contact promotes cooperative behavior. For instance, Meleady et al. (2016) used the prisoner’s dilemma paradigm and found that individuals in positive inter-group contact groups were more likely to cooperate with out-group members subsequently than in control groups. Reimer et al. (2017) found that positive inter-group contact can promote cooperation with out-group members by improving the evaluation of external groups. The contact hypothesis (Allport, 1954) suggested that through positive, equitable, and cooperative interactions, biases and hostilities between different groups can be reduced, thus promoting social harmony and understanding. Social identity theory also supports this view, pointing out that individuals prefer the group they belong to, hold more trust in the members of the internal group, and show higher altruistic tendencies, while they suspect and disparage the external group (Hogg et al., 2017; Eaton, 2011). For example, individuals show a higher propensity to cooperate with in-group members than with out-group members, and even more leniency and weaker punishment are given to in-group members when unfair behavior occurs in cooperation. However, the previous studies on the effect of cross-strait contact often used a self-report method to measure attitude and cooperation intention (Tang, 2017; Zhang, 2017), and because people often cannot turn their will into action, there is an intention-behavior gap (Sheeran, 2002). Using a self-reporting method to investigate individual attitudes and intentions will have certain limitations. Due to historical reasons for cross-straits, the gap between the actual cooperative behavior and the intention of the Taiwanese youth may be more significant (Tang, 2017), and it will be of more practical significance to document the actual cooperative behavior. To this end, this study uses a trust game paradigm to explore the impact of inter-group contact to measure the level of cooperative behavior of young people across the Taiwan straits.

This paper focused on the kind of Taiwanese youth for whom intergroup contact works better.

*H1*: Taiwanese youth exposed to inter-group contact have higher levels of cooperative behavior than those not exposed to inter-group contact.

### 1.2 The mediating role of social distance

Zimer pointed out that social distance measures the inherent spiritual connection between oneself and others, while larger social distance implies an ‘inner barrier’ between individuals. Social distance is used to describe the emotional intimacy and relationship closeness between individuals in a society, reflecting similarities based on social variables or social networks (Charness et al., 2007; Eaton, et al., 2011). In social interactions, it is defined as the degree of intimacy between participants. Prior research suggests that the influence of positive intergroup contact on cooperative behavior is mediated by changes in social distance. For instance, Yuan et al. (2014) found that narrowing social distance leads to a higher tendency for individuals to choose cooperative solutions when investigating the effects of social distance on game cooperation and conflict behavior. In essence, social distance can moderate willingness to cooperate. Interactive participants are more inclined to cooperate with those they have closer social distance to, such as familiar individuals or members of the in-group, as opposed to those with greater social distance, such as strangers or members of out-groups.

Over time, factors such as education, culture, and Taiwanese youth’s limited understanding of mainland Chinese youth have influenced the social distance between cross-strait youth. However, as cross-strait youth exchanges continued to expand, the opportunities and areas for interaction increased, which potentially led to a reduction in social distance among cross-strait youth. Prior research indicated that intergroup contact can narrow social distance. For instance, Wang et al. (2016) discovered that increased contact opportunities allowed urban residents to understand the lifestyles and values of migrant workers more, thus reducing social distance to some extent and dissipating barriers and misunderstandings. Similarly, Guan et al. (2007) explored the impact of social distance on cooperative and conflict behavior, finding that people tended to cooperate more as social distance diminished. Moreover, influenced by the Chinese cultural concepts of righteousness and relational trust, within the context of Chinese culture, the effects of social distance might be more pronounced. In this cultural context, people are more concerned about relationships than interests when dealing with acquaintances. Consequently, when deciding whether to cooperate with mainland Chinese youth, young people in Taiwan are also influenced by the social distance between them.

Given that positive intergroup contact can narrow social distance and enhance intimacy with members of the other group, Taiwanese youth who have studied or worked in the Chinese mainland might be more rational and friendly in their outlook on cross-strait relationships and mainland Chinese youth. As intergroup contact deepens and understanding of the mainland deepens, the social distance between cross-strait youth could improve to some extent, thereby influencing their cooperative behavior.

*H2*: Taiwanese youth who have closer social distance with mainland youth demonstrate higher levels of cooperative behavior after group interactions than larger social distance.

### 1.3 The moderating role of individualism

It is worth noting that the influence of positive inter-group contact on cooperation is also moderated by an individual’s cultural orientation, especially by individualism (Li et al., 2015). Considering that cross-strait people, in a general sense, belong to the same traditional collectivistic culture, the major cultural orientation difference between them is individualism, which can be reflected in the “small happiness” culture in Taiwan. So, individualism will be put at the center of the research paradigm.

Firstly, as a dimension of cultural orientation, individualist orientation reflects specific attitudes on relationships (Grossmann and Na, 2014; Apanovich et al., 2018), which moderates the direct influence of social distance on cooperation. Individualism emphasizes ego and independence. In deciding whether to cooperate, individualists are more concerned about profit, and less influenced by relationships with partners (Apanovich et al., 2018). Individualism will undermine the impact of social distance on cooperation. Secondly, individualism and collectivism diverge in “responsibility for the inner group.” Individualists think for themselves and consider personal interests over group interests (Apanovich et al., 2018). This means profit is the prior factor they use to decide on whether to cooperate. Without profit, the shortened social distance brought about by intergroup contact is not enough to promote the cooperation of people who highly prefer individualism. In other words, individualism may mitigate the direct impact of positive intergroup contact on cooperation.

The individualism of Taiwanese youth is a cognitive framework for negotiating the relationship between self and others.

*H3*: Individualism is a significant moderator in the relationship between positive inter-group contact and cooperation and moderates two paths: The effect of positive inter-group contact on cooperation became stronger in the less individualistic group. The effect of social distance on cooperation became stronger in the less individualistic group.

## 2 Method

### 2.1 Participants

The participants in this study were Taiwanese youth (Mage = 22.78, SDage = 4.18), defined as individuals aged between 18 and 45 years in accordance with the “Youth Guidance Development Law (Draft).” From August 2021 to May 2022, a total of 350 questionnaires were distributed online (i.e., questionnaire stars and social networking sites) and offline (through campus recruitment). After excluding 19 invalid questionnaires with incomplete contents, five questionnaires with excessively short completion times (total time less than 3 min) and four questionnaires with the same answer for each question, 322 valid questionnaires were selected, resulting in an effective recovery rate of 92%. According to G * Power to estimate the expected effect size, 302 valid participants are required when the medium effect size is reached and the explanatory force is 95% (1 – β = 0.95, *ɑ* = 0.05). The effective participants of this study meet the demanded quantity.

The valid participants included undergraduate, master’s, and doctoral students, from more than 10 universities in both the mainland and Taiwan. Of the participants, 57.14% were from universities in Taiwan, e.g., Zhongxing University (*N* = 62) and Taiwan Normal University (*N* = 42), while 42.86% of the participants were from universities in the mainland, e.g., Xiamen University (*N* = 8) and Beijing Normal University (*N* = 6). The mean age was 22.78 years old (SD = 4.18), of which 145 were men (45.03%) and 177 women (55.97%). The proportion of participants with more than half a year of mainland experience was 42.2 percent. Participants were involved in the trust game after completing the questionnaires.

### 2.2 Measurements

#### 2.2.1 Positive inter-group contact scale

The positive intergroup contact questionnaire was developed by Dhont et al. (2011) and consists of four items. The total score represents the overall situation of the participant’s positive intergroup contact. Sample items include “How often do you have pleasant contact with mainland youth?” and “How often do you have positive experience [*sic*] with mainland youth until now?” The scale uses a Likert-type 7-point scale, ranging from “1 = seldom” to “7 = almost everyday.” The higher the score, the more positive the intergroup contact. The results of the validation factor analysis showed that the overall fitting index of the questionnaire was χ^2^/df = 6.48, NFI = 0.963, IFI = 0.964, CFI = 0.964, and RMSEA = 0.076. According to Wen et al. (2004), the fitted parameter values of each variable were within the ideal range (IFI > 0.8; NFI > 0.8; CFI > 0.8), indicating that the questionnaire had good structural validity. The factor loading of each question item was [0.53, 0.76], and the Cronbach coefficient of the questionnaire was 0.83.

#### 2.2.2 Individualism–collectivism scale

The individualism–collectivism scale was developed by Triandis and Gelfand (1998) and consists of 16 items that can be divided into two dimensions: individualism and collectivism. Questions 1 ~ 8 are used to measure individualism orientation, e.g., I spend most of the time relying on myself, rarely on others; questions 9 ~ 16 are used to measure collectivism orientation, e.g., I like to work with others. The scale uses a Likert-type 7-point scale, ranging from “1 = strongly disagree” to “7 = strongly agree.” The higher the score, the more pronounced the individual has this tendency. The results of the validation factor analysis showed that the overall fitting index of the questionnaire was χ^2^ / df = 4.36, NFI = 0.851, IFI = 0.912, CFI = 0.869, and RMSEA=0.082. According to Wen et al. (2004), the fitted parameter values of each variable were within the ideal range (χ^2^ / df < 5; IFI > 0.8; NFI > 0.8; CFI > 0.8), the KMO value was 0.816, and the Bartlett sphericity test was 0.000. This indicated that the questionnaire had good structural validity, the factor loading of each item was [0.36, 0.85], the Cronbach coefficient of the questionnaire was 0.80.

#### 2.2.3 Social distance scale

The Social Distance Scale was revised from the social distance scale by Bastian et al. (2012); it consists of six items and can be divided into six dimensions: schoolmate, classmate, friend, close friend, neighbor, and husband/wife. Examples are as follows: “I would enjoy having a mainland friend,” “I would enjoy having a close mainland friend,” and “I would marry a mainland people”[*sic*]. The scale uses a Likert-type 5-point scale, ranging from “1 = strongly disagree to 5 = strongly agree.” The higher the score, the closer social distance people have with mainland youth. The KMO value was 0.829, and the Bartlett sphericity test was 0.000. The Cronbach coefficient of this scale in this study was 0.81.

#### 2.2.4 Trust game

The “trust game”(Corgnet et al., 2016) was used to measure the tendency to cooperate with mainland youth. “The experimental procedure is designed with reference to Bayesian models, incorporating prior knowledge to simulate decision-making behavior in trust games between Taiwanese and mainland youth in real-world scenarios. The prior probability distribution Pr represents participants’ pre-information beliefs, reflecting the initial inclination of Taiwanese youth toward ‘equally sharing’ (*μ* = 0.5, *σ* = 0.2). The posterior probability distribution P represents participants’ post-information beliefs about the state of the environment, indicating the tendency of Taiwanese youth to share equally based on factors like reciprocity and risk preference. The formation of posterior beliefs at time t is contingent upon the prior beliefs Pr at that moment, the observed set of interactive behaviors Ot (feedback given by mainland youth in previous rounds, i.e., whether they share 100 yuan with the other player), and the reward set Rt, collectively influencing the decision-making process.”

The participants entered the formal experiment after three exercises. Before the game, the participants were told, “You will play a game with a mainland youth. At the beginning of the game, you have ¥1,000. In the following 15 choices, you can choose whether to invest ¥100. If you choose to invest, the mainland youth will not only get back ¥100 of his original investment but get an extra ¥500. If the other player chooses to share the income equally, you will get ¥300; otherwise, if the other player does not choose to share equally, you will lose ¥100. Correspondingly, if you choose not to give, you will get ¥100.” Before the game started, participants were told to get as much money as possible in the game, and the final amount of money in the game would be positively related to the reward they got after the experiment. The reward is 1.5% of the final amount in the trust game. The level of cooperation was measured by the number of times the participants chose to give money to the mainland youth in the 15 choices of trust game.

### 2.3 Harman single-factor test

Data for this study were collected by self-report and, therefore, were tested for common methodological bias (CMB) by means of the Harman single-factor test before data analysis (Podsakoff et al., 2003; Wen et al., 2004). The Harman single-factor method often uses exploratory factor analysis to test the CMB. EFA suggests that there is a method factor that explains the common variation across all items of a study with different traits (Wen et al., 2004). The more variation explained by method factors, the more serious the bias. Podsakoff and Organ (1986) suggested that the single-factor explanatory variation obtained by EFA (unrotated) did not exceed 50% and that CMB was not severe. According to the application in the country, it is generally believed that the variation explained by a single factor cannot exceed 40%. But whether it is 50 percent or 40 percent, it is all about empirical criterion. These evaluation criteria are not based on strict theories or scientific formulas but rather derived from extensive empirical research and practical experience. Therefore, there may be some flexibility and variability in different contexts. The results show that six factors with characteristic roots greater than 1 were obtained without rotation, explaining 72.75% of the variation, and the first common factor explained 23.10% of the total variation and less than 40% of the critical criterion, indicating that there is no serious common methodological bias problem in this study.

## 3 Data analysis and results

### 3.1 Description and correlation analysis of positive inter-group contact, social distance, individualism, and cooperation

There are significant correlations between positive inter-group contact and social distance (*r* = 0.577, *p* < 0.01, 95% CI [0.514, 0.653]) and cooperation (*r* = 0.558, *p* < 0.01, 95% CI [0.477, 0.616]). There are significant correlations between social distance and cooperation (*r* = 0.453, *p* < 0.001, 95% CI [0.343, 0.530]). There are significant correlations between cooperation and individualism (*r* = −0.262, *p* < 0.05, 95% CI [−0.381, −0.206]) and collectivism (*r* = 0.322, *p* < 0.001, 95% CI [0.253, 0.454]) (Table 1).

**Table 1:** Mean, standard deviation, and correlation coefficient of each variable.

Variable	M	SD	1	2	3	4	5	6
1.Age	22.78	4.18	1					
2.Educational background	1.84	0.53	0.434^**^					
3.Positive inter-group contact	16.57	6.75	0.20^**^	−0.237^*^	1			
4.Social distance	31.98	4.70	0.132^*^	−0.141^*^	0.577^***^	1		
5.Individualism	40.53	4.96	−0.214^*^	−0.138^*^	−0.064	−0.039	1	
6.Cooperation	4.77	1.42	−0.040	−0.205^*^	0.558^***^	0.453^***^	−0.262^**^	1

^*^p < 0.05; ^**^p < 0.01, ^***^p < 0.001.

### 3.2 The mediating role of social distance

Firstly, to test the mediating role of social distance between positive intergroup contact and cooperation, version 23.0 of SPSS and version 3.4 of PROCESS (Model 4) were used.

The results showed that the direct effect of positive intergroup contact on cooperation was significant (*β* = 0.396, *p* < 0.001). Taiwanese youth with positive intergroup contacts show higher cooperation. Taiwanese youth exposed to intergroup contact have higher levels of cooperative behavior than those not exposed to inter-group contact. H1 was supported. The inclusion of social distance into the regression equation revealed that positive intergroup contact had a significant effect on social distance and social distance had a significant effect on cooperation; the mediating effect was significant (*β* = 0.23, *p* < 0.01), demonstrating that social distance has a mediating effect between positive intergroup contact and sleep cooperation, with the mediating effect accounting for 18.3% of the total effect (0.126; Table 2). Taiwanese youth who have closer social distance with mainland youth demonstrate higher levels of cooperative behavior after group interactions than larger social distance (*p* < 0.01). H2 was supported.

**Table 2:** Mediating effect of social distance.

Model pathway	Coefficient	Standardized error	*t*-value	*p*-values
Direct effect				
Social distance				
Intergroup contact	0.396	0.030	12.99	<0.001
Cooperation				
Social distance	0.059	0.013	3.05	<0.01
Intergroup contact	0.103	0.03	7.86	<0.001
Indirect effect	0.023			<0.001
Total effect	0.126			<0.001

### 3.3 The moderating role of individualism–collectivism

Secondly, the moderating role of individualism–collectivism was tested. Version 23.0 of SPSS and version 3.4 of PROCESS (model 15) were used to perform the analysis. In this study, the first stage estimated the moderating role of individualism–collectivism in the relationship between the independent variable (positive inter-group contact) and the dependent variable (cooperation), and the second stage tested the moderating role of individualism–collectivism in the relationship between the mediating variable (social distance) and the dependent variable (cooperation level). In a hierarchical regression equation, individualism–collectivism and positive intergroup contacts and their cross-terms are centralized. According to Muller et al. (2005), if the model estimates meet, in the first stage, the independent variable affects the mediating variable, and the independent variable and the adjusting variable are significant to the dependent variable. It means that there is a moderated effect.

As shown in Table 2, in the stage 1 regression equation, the positive inter-group contact positively predicted the social distance (*β* = 0.40, SE = 0.03, *p* < 0.001). The regression equation result of stage 2 showed that the interaction term of positive inter-group contact and individualism can positively predict cooperation (*β* = −0.01, SE = 0.003, *p* < 0.05), and the interaction term of individualism and social distance can negatively predict cooperation (*β* = −0.01, SE = 0.004, *p* < 0.05). Moreover, social distance can positively predict the cooperation (*β* = 0.41, SE = 0.14, *p* < 0.01). The results showed that individualism plays a moderating role in the relationship between positive inter-group contact and cooperation and moderates the two paths: The effect of positive inter-group contact on cooperation became stronger in the less individualistic group. The effect of social distance on cooperation became stronger in the less individualistic group (Table 3). H3 is supported.

**Table 3:** Test of the moderating role of individualism–collectivism (*n* = 322).

		Step 1: social distance		Step 2: cooperation	
		*β*	SE	*β*	SE
Controlled variable	Age	0.024	0.06	−0.015	0.02
	Educational background	−0.120	0.48	−0.039	0.15
Predictive variable	Positive inter-group contact	0.392^***^	0.03	0.303^***^	0.10
	Individualism			−0.278^**^	0.10
	Collectivism			−0.166	0.14
	Social Distance			0.406^**^	0.14
	Positive inter-group contact × Individualism			−0.01^*^	0.003
	Social Distance × Individualism			−0.01^*^	0.004
	∆R^2^	0.346^***^		0.432^***^	
	F	55.99		34.06	

### 3.4 The effect of positive inter-group contact on cooperation under different levels of individualism

In order to reveal the moderating effect of individualism in two paths more clearly, we tested the effect of positive inter-group contact on cooperation under different levels of individualism and performed a simple slope test. We divided individualism into three levels—low (mean minus one standard deviation), mid (mean), and high (mean plus one standard deviation)—and tested the direct effect of positive inter-group contact on cooperation under three levels of individualism. The results showed that positive inter-group contact was significant (low = 0.10, SE = 0.02, SE = 0.02; middle = 0.0.001, SE = 0.02, *p* < 0.01), and positive inter-group contact did not affect cooperation in the case of high individualism.

**Figure 1:**
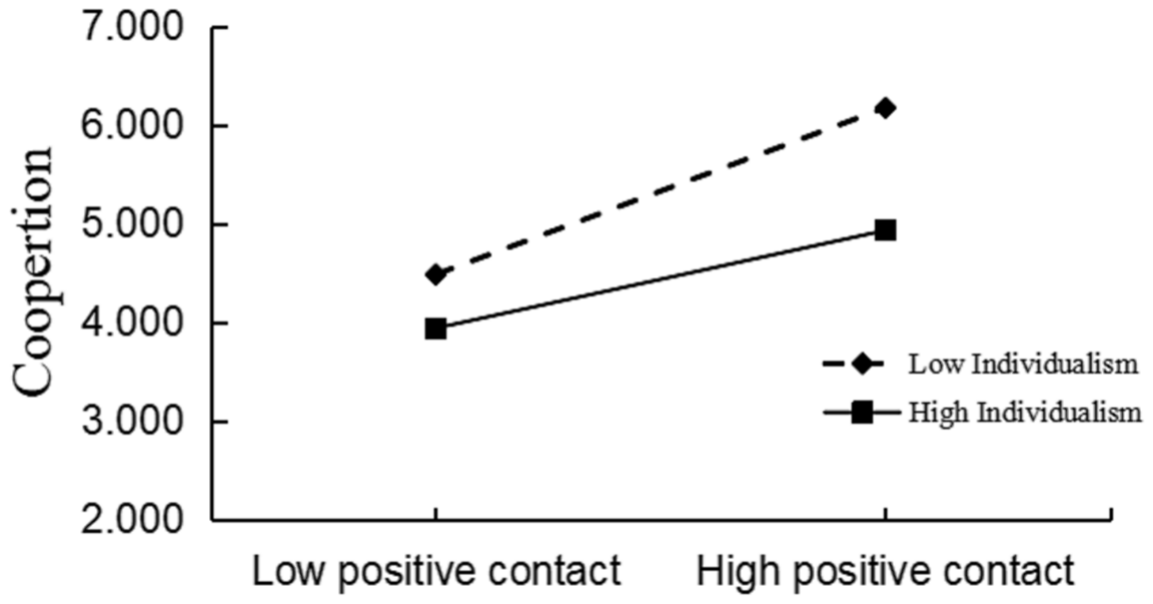
The moderating role of individualism in the relationship between positive inter-group contact and cooperation. *p<.05, **p<.01, ***p<.001.

Simple slope tests showed that for low-individualism Taiwanese youth, the effect of positive inter-group contact on cooperation was significant (*simple slope* = 0.12, *t* = 4.32, *p* < 0.001); for high-individualistic Taiwanese youth, the effect of positive inter-group contact on cooperation was not significant (*simple slope* = 0.08, *t* = 0.56, *p* > 0.05). Table 4 presents the direct effect under three levels of individualism. In order to emphasize the significant differences in extreme scenarios, Figure 1 only compared the high and low extremes making it clearer to demonstrate the notable impact of cluster contact on cooperation in situations of extreme individualism. Compared with high-individualism Taiwanese youth, positive inter-group contact has a more obvious positive effect on low-individualism Taiwanese youth (see Figure 1).

**Table 4:** The direct effect of positive inter-group contact on cooperation under different levels of individualism.

	Individualistic level	Effect	*SE*	*t*
Low individualism (*n* = 112)	35.39	0.10	0.02	4.32^***^
Mid individualism (*n* = 137)	40.34	0.05	0.02	3.19^**^
High individualism (*n* = 73)	45.29	0.02	0.03	0.56

^*^p < 0.05; ^**^p < 0.01, ^***^p < 0.001.

Next, the Bootstrap test of the regression model was done using the program PROCESS of SPSS. To examine the indirect effects of positive intergroup contact on cooperation under different levels of individualism, we divided individualism into three levels: low (mean minus one standard deviation), mid (mean), and high (mean plus one standard deviation). We tested the direct effect of positive inter-group contact on cooperation under three levels of individualism. The results showed that (Table A1) at low levels of individualism, the mediating effect of social distance is significant (95% CI [0.019, 0.063]). The effect size was 0.04; for the level of moderate individualism, the mediating effect of social distance was significant (95% CI [0.007, 0.041]), and the effect size was 0.02. However, under the high individualism level, the mediating effect of social distance was not significant. This shows that with individualism increasing, the mediating role of social distance gradually weakens.

**Figure 2:**
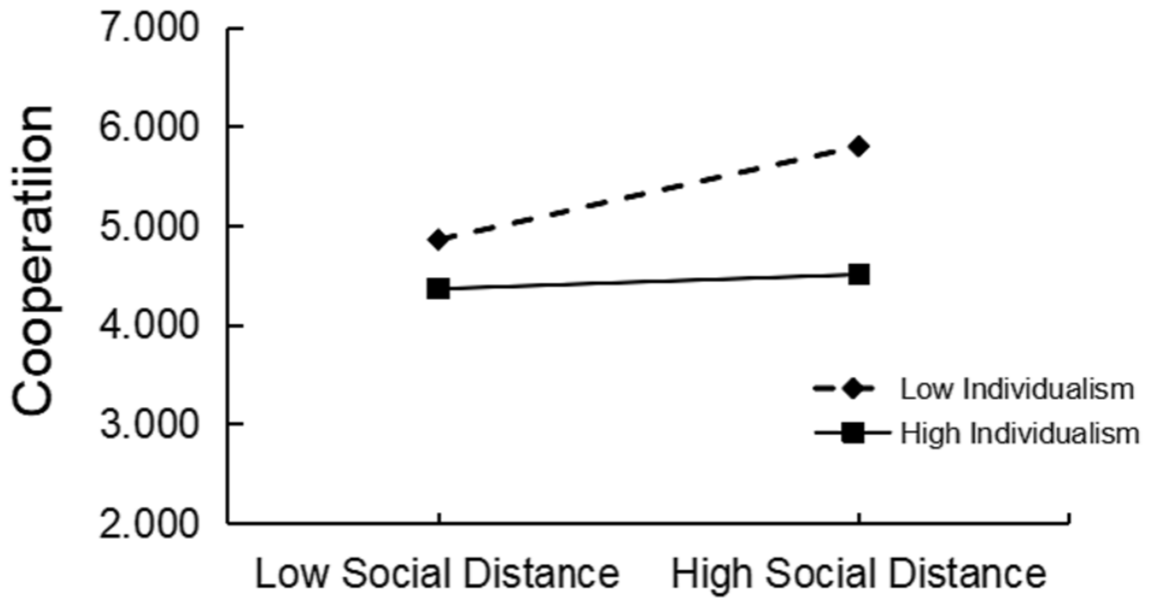
The moderating role of individualism on the relationship between social distance and the level of cooperation.

To clearly show the moderating role of individualism between social distance and cooperation, an interaction map is also drawn (see Figure 1). In general, cooperation increases as the social distance gets closer. However, for high-individualism Taiwanese youth, there was no significant difference between cooperation with a low social distance player and a high social distance player (*simple slope* = 0.02, *t* = 0.566, *p* > 0.05). In addition, the level of cooperation among Taiwanese youth with low individualism was always higher than that of high individualism, indicating that individuals have a higher preference for cooperation (Figure 2).

## 4 Discussion

### 4.1 The effectiveness of positive inter-group contact in promoting cooperation

The results show that positive inter-group contact has a significant positive effect on improving cooperation among cross-strait youth. Taiwanese youth with more positive inter-group contact showed higher cooperation with mainland youth. Promoting positive inter-group contact is an effective way to improve the cross-strait relationship. The results are consistent with previous literature exploring the effect of positive intergroup contact. Positive intergroup contact has been shown to be an effective way to promote cooperation between the two groups (Kauff et al., 2017; Reimer et al., 2017; Meleady et al., 2016). The results also show social distance and individualism have a significant impact on the effect of positive inter-group contact.

For a long time, under the influence of public opinion, educational institutions, and the media, some Taiwanese youth have developed certain negative initial impressions of and prejudices against mainland youth. For example, Tang (2017) pointed out that Taiwanese youth groups have prejudices against the mainland. Due to historical reasons and the policy restrictions of the Taiwan authorities, some Taiwanese youth have a relatively large social distance from mainland youth and have shown lower cooperation willingness (Appendix Table 2). As the level of positive inter-group contact increases, the positive emotions generated in inter-group contact, such as appreciation, can effectively improve attitudes toward mainland youth and narrow the social distance with mainland youth. The mediating effect of social distance directly affects cooperation between Taiwanese youth and mainland youth.

### 4.2 The moderating role of individualism

Firstly, this study reveals that Taiwanese youth with low levels of individualism are the most receptive audience for cross-strait cluster contact. After intergroup contact, they exhibit social proximity closer to mainland youth, along with higher levels of cooperative behavior.

**Figure 3:**
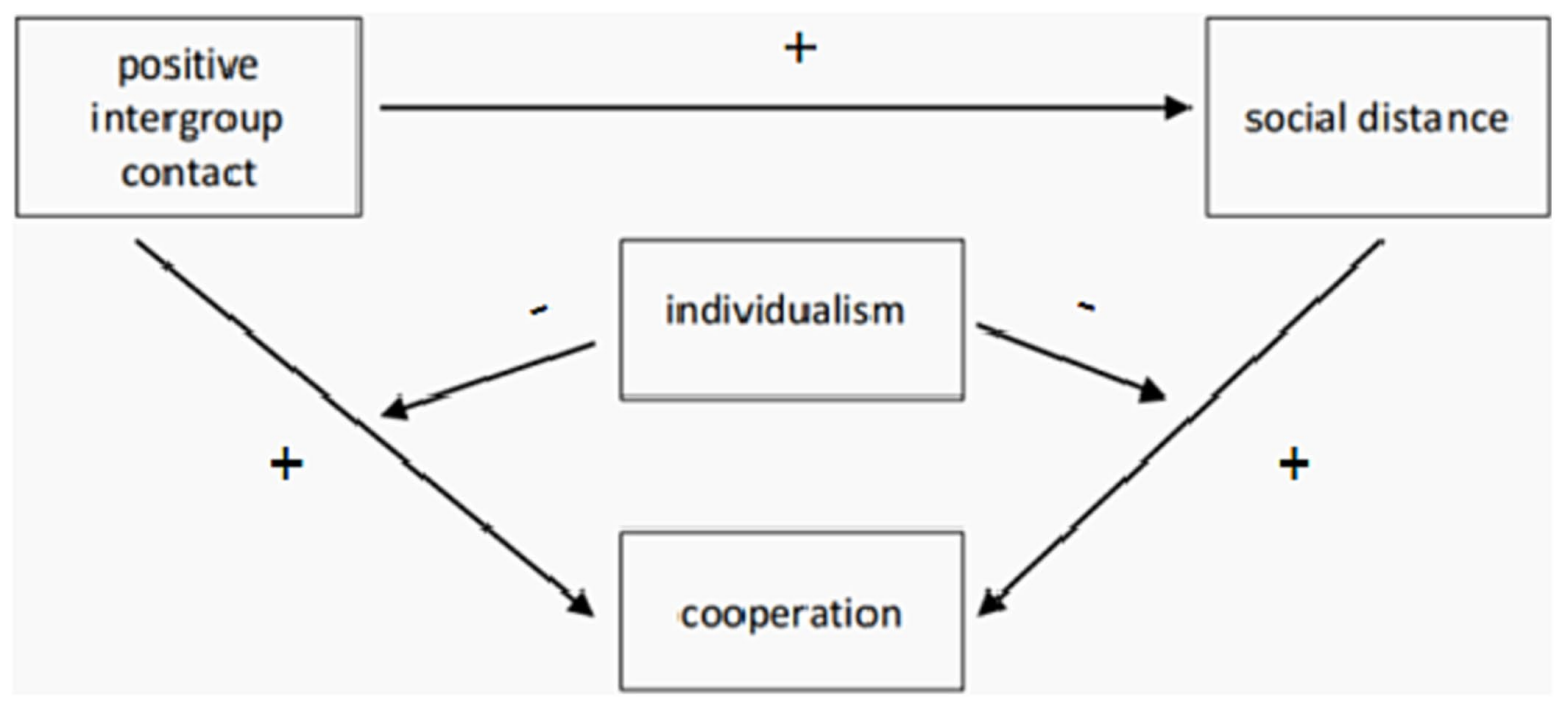
Hypothtical model of intergroup contact, social distance and cooperation.

Besides, The present study found that individualism moderated the relationship between positive inter-group contact and cooperation through two paths. Firstly, individualism moderated the direct influence of positive intergroup contact on cooperation. Although positive inter-group contact positively predicted cooperation, the effect was more significant in low-individualism Taiwanese youth than that of high-individualism Taiwanese youth (see Figure 3). As the present study used a trust game to measure cooperation, the cooperation in this study reflected cooperation based on trust.

The results were a bit different from previous studies (Scarpati and Pina, 2017), which suggested that individualists emphasize personal benefit and competition, advocate individual goals, and interests overweigh group goals, which are more likely to be influenced by training. However, as the effect of positive intergroup contact on cooperation is mainly based on narrowing the psychological distance and promoting trust between the two groups (Brewer, 1996; Wang and Mao, 2016), it has less influence on individualists who emphasize free choice and personal interest. Positive intergroup contact may not be a good training sufficient to improve high-individualism Taiwanese youth’s cooperation. Besides, individualism emphasizes personal interests and achievements (Grossmann and Na, 2014); whether they can profit from cooperation is what interests Taiwanese youth with high individualism more. Since the reward won by the participants in the present study is 1.5% of the final amount in the trust game (¥15–¥25), it is not tempting enough to entice them to cooperate with mainland youth, and the cooperation of high-individualism Taiwanese youth does not increase much..

Additionally, individualism plays a moderating role in the relationship between social distance and cooperation. The effect of social distance on cooperation is significant for low-individualism Taiwanese youth (Figure 1), which was consistent with previous studies. According to the self-construction theory (Sedikides et al., 2013), individualism emphasizes independence, uniqueness, and free choice, while collectivism emphasizes relationship and social embeddedness (Grossmann and Na, 2014). Low-individualism Taiwanese youth tend to be more relationship-oriented than profit-oriented (Han and Yue, 2009), and they tend to cooperate more when playing games with players at a close social distance.

In addition, the overall cultural orientation of Taiwanese youth deserves attention. Previous studies focusing on individual/collectivism among Taiwanese youth have shown inconsistent results. The study by Tu et al. (2012) pointed out that Taiwanese youth have higher collectivism; the study by Zhang (2017) pointed out that Taiwanese youth showed higher individualistic tendencies. The authors believe that this is due to the different age definitions of the youth group. Influenced by the trend of globalization and post-materialism in Taiwan in recent years, the younger generation in Taiwan shows a higher level of individualism. This study also shows that the age and education of Taiwanese youth are also related to the level and effect of positive inter-group contact. Exploring the overall cultural orientation of Taiwanese youth will provide theoretical support for deepening cross-strait youth exchanges and cooperation.

In summary, this study constructed a mediating model to examine the moderating effect of individualism on the mediating process of “positive inter-group contact → social distance → cooperation.” The results show that social distance is a significant mediator between positive inter-group contact and cooperation, and individualism is a significant moderator. This helps to deeply understand the relationship between positive inter-group contact and cooperation between cross-strait youth and their internal mechanism.

Therefore, in the future, cross-strait youth contact activities should focus on how to create a good intergroup contact atmosphere that promotes status equality and friendship and focus on high-individualism Taiwanese youth in order to improve the effectiveness of intergroup contact between cross-strait youth.

### 4.3 Conclusion

This paper dives into Taiwanese youth with different cultural beliefs and finds that (1) positive inter-group contact can significantly promote cooperation between Taiwanese youth and mainland youth. (2) Social distance plays a mediating role between intergroup contact and cooperation. Taiwanese youth who have closer social distance with mainland youth demonstrate higher levels of cooperative behavior after group interactions than larger social distance. (3) Individualism has a significant moderating role in the relationship between positive inter-group contact and cooperation and in the relationship between social distance and cooperation. The effect of positive inter-group contact on cooperation became stronger in the less individualistic group. The effect of social distance on cooperation became stronger in the less individualistic group.

### 4.4 Limitations and future research directions

Although some valuable results have been obtained in this study, there are still some deficiencies worth noting, which need to be improved in further research. For example, this study uses the trust game paradigm to measure the level of cooperation between Taiwanese youth and mainland youth. Affected by the framing effect, there may be differences in the cooperation behaviors of young people on both sides of the Taiwan Strait in some decision-making situations. Besides, this study used the scale by Triandis and Gelfand (1998) to measure the cultural beliefs of Taiwanese youth because this study is eager to know the differences between cross-strait youth from the group level, but using the self-construal concept, i.e., independent and interdependent self, will help to understand the differences from an individual level. According to the framework effect, an individual’s behavior is influenced by both personality and the environment, and an individual’s cultural orientation can be influenced by the environment. Future research could focus on the role of cultural contexts. Using diverse paradigms to explore the level of cooperation between cross-strait youth and supplementing the results of this study will help to better understand the role of positive inter-group contact on cooperation.

## Data availability statement

The original contributions presented in the study are included in the article/supplementary material, further inquiries can be directed to the corresponding author.

## Ethics statement

The studies involving humans were approved by the survey and Behavioral Research Ethic Committee of Xiamen University. The studies were conducted in accordance with the local legislation and institutional requirements. The participants provided their written informed consent to participate in this study.

## Author contributions

RKX: Formal analysis, Project administration, Resources, Supervision, Writing – review & editing. SXL: Formal analysis, Investigation, Methodology, Software, Writing – original draft.

## Appendix

**Table A1:** The mediating role of social distance at different levels of individualism.

	Individualistic level	Effect	*Boot SE*	*BootLLCI*	*BootULCI*
Low individualism (*n* = 112)	35.39	0.04	0.011	0.019	0.063
Mid individualism (*n* = 137)	40.34	0.023	0.008	0.007	0.041
High individualism (*n* = 73)	45.29	0.006	0.012	−0.016	0.029

**Table A2:** Intergroup contact with mainland youth.

	Never/rarely		Occasionally		Medium		Frequent		Almost everyday	
	Frequency	Percentage	Frequency	Percentage	Frequency	Percentage	Frequency	Percentage	Frequency	Percentage
Pleasant contact	45	13.9%	96	29.8%	34	10.6%	93	28.9%	14	4.3%
Positive experience	41	12.7%	100	31.1%	31	9.6%	97	30.1%	13	4.3%
Contact with mainland youth	52	16.2%	91	28.3%	32	9.9%	37	11.5%	70	21.7%
Communication with mainland youth	43	13.4%	131	40.6%	7	2.1%	64	19.9%	37	11.5%
